# The complete mitochondrial genome of *Dermacentor* (*Indocentor*) *auratus* (Acari, Ixodidae)

**DOI:** 10.1051/parasite/2021002

**Published:** 2021-01-19

**Authors:** Jean-Marc Chavatte, Sophie Octavia

**Affiliations:** National Public Health Laboratory, National Centre for Infectious Diseases Block G, Level 13, 16 Jalan Tan Tock Seng Singapore 308442 Singapore

**Keywords:** *Dermacentor* (*Indocentor*) *auratus*, Mitochondrial genome, Long-range PCR, Illumina sequencing

## Abstract

*Dermacentor* (*Indocentor*) *auratus* Supino, 1897 is a prominent ixodid vector of numerous pathogens of public health and veterinary importance. Using long-range PCR of two overlapping regions sequenced on an Illumina MiSeq machine, the complete mitochondrial genome of *D. auratus* is reported here. The resulting contigs were able to be assembled into a complete and circularised genome which had the general organisation of the mitochondrial genomes of the Metastriates. It had a total length of 14,766 bp and contained 37 genes, including 13 protein-coding genes, 22 transfer RNA genes, and 2 ribosomal RNA genes, as well as 2 non-coding control regions and 3 tick-boxes. The phylogenetic analysis on the whole mitogenome confirmed the position of *D. auratus* within the *Dermacentor* clade.

## Abstract (fr)

*Dermacentor* (*Indocentor*) *auratus* Supino, 1897 est un ixode, vecteur notable de nombreux pathogènes importants en santé publique et en médecine vétérinaire. Utilisant la PCR en fragment long sur deux régions chevauchantes combinée au séquençage sur une plate-forme Illumina MiSeq, le génome mitochondrial complet de *D. auratus* est présenté ici. Les contigs obtenus ont été assemblés en un génome circulaire complet conforme à l’organisation générale des génomes mitochondriaux des Métastriates. D’une longueur totale de 14766 pb, il contient 37 gènes, incluant 13 gènes codants des protéines, 22 gènes d’ARN de transfert et 2 gènes d’ARN ribosomaux, ainsi que 2 régions contrôles non-codantes et 3 motifs « tick-box ». L’analyse phylogénétique sur le mitogénome complet confirme la position de *D. auratus* au sein du clade *Dermacentor*.

## Introduction

Ticks are the second largest pathogen transmitters globally, after mosquitoes [7], and tick-borne diseases (TBDs) have a colossal impact on livestock industries worldwide, and also constitute an important public health threat for humans [20]. The recent increase in TBD infections in the United States and globally, added to the increase travel fluxes and mobility, accentuates the risk of import of TBDs into new areas, as recently highlighted with the first imported case of human babesiosis in Singapore [15]. Following import, the risk of introduction and establishment of a TBD locally depends on the vector competency of the local tick populations. It is therefore important to inventory the tick fauna, particularly in areas where this has been overlooked or neglected [13] to perform risk assessments. Renewed interest in this field has lead to interesting reports such as the recent discovery of *Dermacentor* (*Indocentor*) *auratus* Supino, 1897 [24] in Singapore, from multiple hosts, including humans [14]. *Dermacentor auratus* is a competent vector of the highly pathogenic zoonotic Kyasanur Forest disease (KFD) virus [21] and the murine Lanjan Virus [25]. Several other pathogens have also been isolated from *D. auratus* such as *Anaplasma* spp., *Rickettsia* spp., *Francisella* spp., *Borrelia* spp. and *Hepatozoon* spp. [22, 23, 27], highlighting the veterinary and public health risks related to this species [14, 22, 23, 27].

Along with the report of the discovery of *D. auratus* in Singapore, preliminary molecular data on its mitogenome were provided with sequences of the cytochrome c oxidase I (*cox1*) and the large subunit ribosomal RNA (*lsu rRNA*) genes [14]. The sequences were deposited in GenBank under accession numbers MT371767 and MT371768 (*cox1*) and MT371591 and MT371592 (*lsu rRNA*), respectively [14] and were used as the starting point for the present work that reports the entire mitochondrial genome of *D. auratus*.

## Materials and methods

DNA material from a *D. auratus* TKL1 isolate obtained in [14] was used. Assuming that the mitogenome of *D. auratus* is circular, similar to other Metastriate ticks, its amplification was attempted using two long-range PCRs targeting fragments of ≈9700 bp and ≈6200 bp spanning the whole, and overlapping at their extremities by 457 bp and 709 bp. The primers proposed by [4] and [9] and successfully used in [14] were cross-paired. The PCRs were run in a total volume of 30 μL containing 1X High Fidelity PCR Buffer, 2 mM of MgSO_4_, 250 μM of each dNTP, 250 ηM of each oligonucleotide primer, 0.06 U/μL of Platinum^®^ *Taq* High Fidelity DNA Polymerase (Invitrogen™) and 3 μL of DNA template. The PCR assays were performed on a Veriti thermal cycler (Applied Biosystems), with the following conditions: initial denaturation at 95 °C for 2 min, followed by 35 cycles of annealing at 55 °C for 1 min, extension at 68 °C for 6 or 10 min depending on the fragment size (increased by 20 s at each cycle from the 11th cycle), and denaturation at 94 °C for 30 s, then terminated with a final cycle of annealing at 55 °C for 2 min and extension at 72 °C for 15 min.

PCR products were visualised by capillary electrophoresis on a QIAxcel^®^ Advanced System (QIAgen^®^) equipped with a QIAxcel^®^ DNA High Resolution Kit (QIAgen^®^), then purified using a QIAquick^®^ PCR Purification Kit (QIAgen^®^), eluted in EB and stored at −30 °C until sequencing. Both fragments were prepared for next-generation sequencing (NGS) by a Nextera XT DNA Library Preparation Kit (Illumina™) and sequenced on a MiSeq System (Illumina™) to generate 300 bp paired-end reads. Fastq raw reads were trimmed using Trimmomatic v.0.36 [5] to remove low-quality bases and assembled with Shovill v.1.0.4 with SPAdes as the assembler [1]. Contigs were aligned and scaffolded using the overlapping fragments to reconstruct the full mitogenome of *D. auratus* using CLC Main Workbench v8.0.1 (QIAgen^®^). Annotation was performed using MITOS [3] and manual search of homology and comparison. Transfer RNA secondary structure prediction was obtained using the Vienna RNA Websuite [10]. Multiple alignments of the *D. auratus* mitochondrial genomes obtained in this study with several mitogenomes (39 Ixodidae, 1 Argasidae and 1 Nuttalliellidae) retrieved from GenBank were performed with MUSCLE [8]. Phylogenetic analysis, performed with MEGAX [12], was inferred by the maximum likelihood (ML) method based on the GTR + Γ + I model of evolution [26]. The most appropriate model of nucleotide substitution was selected based on BIC score [19]. Reliability of tree topology, branch support and nodal robustness were assessed by non-parametric bootstrap using 1000 replicates.

The mitochondrial genome sequence obtained in this study was deposited in GenBank under accession number MW034677.

## Results and discussion

**Figure 1:**
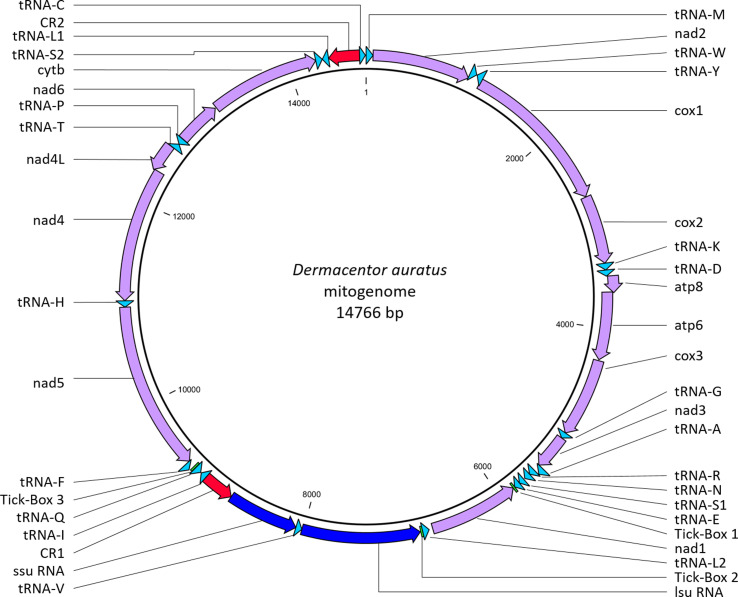
Map of the mitochondrial genome of *Dermacentor* (*Indocentor*) *auratus*. Protein coding genes in purple; ribosomal RNA genes in dark blue; transfer RNA genes in light blue; control regions in red, and tick-boxes in green.

Long-range PCRs, sequencing, and *de novo* assemblies generated 2 contigs of 9700 bp and 6194 bp, respectively with an average sequencing depth of 25- and 44-fold coverage, respectively. After reconstruction, the complete mitogenome of *D. auratus* was confirmed to be circular and showed a total length of 14,766 bp, with AT/GC contents of 77.2% and 22.8%, respectively. Genome annotation revealed homology to the other hard-tick mitochondrial genomes, where it contained 37 genes, including 13 protein-coding genes (*cox1* to *cox3*, *nad1* to *nad6*, *nad4L*, *atp6*, *atp8* and *cytb*), 22 transfer RNA genes (*tRNA*s), 2 ribosomal RNA genes (*rRNA*s), as well as 2 non-coding control regions (CR) and 3 tick-boxes. Details about the organisation, orientation and size of the genes and non-coding elements are listed in Table 1 and mapped in Figure 1.

**Table 1:** Mitochondrial genome organisation of *Dermacentor auratus* with gene order, positions, lengths, nucleic acid composition, coding strand, and start and stop codons.

Gene	Position		Length (bp)	Nucleic acid		Coding strand	Codon		Amino acid size
	Start	End		AT (%)	GC (%)		Start	Stop	
*tRNA-M*	1	68	68	75	25	+			
*nad2*	69	1028	960	83.7	16.3	+	ATT	TAA	319
*tRNA-W*	1027	1088	62	82.3	17.7	+			
*tRNA-Y*	1094	1157	64	73.4	26.6	−			
*cox1*	1150	2688	1539	69.7	30.3	+	ATT	TAA	512
*cox2*	2693	3365	673	71	29	+	ATG	T–	224
*tRNA-K*	3366	3432	67	70.1	29.9	+			
*tRNA-D*	3433	3492	60	78.3	21.7	+			
*atp8*	3493	3654	162	84.6	15.4	+	ATT	TAA	53
*atp6*	3648	4313	666	79	21	+	ATG	TAA	221
*cox3*	4324	5101	778	73.8	26.2	+	ATG	T–	259
*tRNA-G*	5102	5162	61	90.2	9.8	+			
*nad3*	5163	5507	345	80.9	19.1	+	ATT	TAA	114
*tRNA-A*	5507	5570	64	79.7	20.3	+			
*tRNA-R*	5575	5634	60	73.3	26.7	+			
*tRNA-N*	5635	5697	63	76.2	23.8	+			
*tRNA-S1*	5710	5765	56	82.1	17.9	+			
*tRNA-E*	5766	5825	60	78.3	21.7	+			
Tick Box 1	5826	5843	18			−			
*nad1*	5844	6762	919	76.9	23.1	−	ATT	T–	306
*tRNA-L2*	6763	6828	66	72.7	27.3	−			
Tick Box 2	6829	6846	18			−			
*lsu rRNA*	6852	8017	1166	81.6	18.4	−			
*tRNA-V*	8018	8078	61	77	23	−			
*ssu rRNA*	8079	8778	700	78.7	21.3	−			
CR1	8779	9089	311	65.9	34.1	−			
*tRNA-I*	9090	9152	63	73	27	+			
*tRNA-Q*	9160	9225	66	84.8	15.2	−			
Tick Box 3	9229	9246	18			+			
*tRNA-F*	9257	9315	59	86.4	13.6	−			
*nad5*	9315	10,973	1659	79.4	20.6	−	ATT	TAA	552
*tRNA-H*	10,974	11,035	62	83.9	16.1	−			
*nad4*	11,036	12,350	1315	78.7	21.3	−	ATG	T–	438
*nad4L*	12,344	12,619	276	81.5	18.5	−	ATG	TAA	91
*tRNA-T*	12,622	12,682	61	85.2	14.8	+			
*tRNA-P*	12,683	12,743	61	80.3	19.7	−			
*nad6*	12,746	13,180	435	83.2	16.8	+	ATA	TAA	144
*cytb*	13,192	14,273	1082	73.9	26.1	+	ATG	TA−	360
*tRNA-S2*	14,274	14,338	65	81.5	18.5	+			
*tRNA-L1*	14,340	14,400	61	72.1	27.9	−			
CR2	14,401	14,704	304	66.8	33.2	−			
*tRNA-C*	14,705	14,761	57	78.9	21.1	+			

The complete and annotated mitochondrial genome of *D. auratus* has been deposited in GenBank under the accession number MW034677.

### Protein coding genes

Six of the protein-coding genes were initiated with an ATG codon (*cox2*, *atp6*, *cox3*, *nad4*, *nad4L* and *cytb*), six with an ATT codon (*nad1, nad2*, *cox1*, *atp8*, *nad3* and *nad5*), and one with an ATA codon (*nad6*). Eight of the protein-coding genes were terminated with the standard TAA codon (*nad2*, *cox1*, *atp8*, *atp6*, *nad3*, *nad5*, *nad4L* and *nad6*), and five with the truncated termination codons TA− (*cytb*) and T− (*cox2*, *cox3*, *nad1* and *nad4*). Alternative start codons and truncated stop codons T− or TA− have been found in the mitogenomes of other *Dermacentor* species [11], other ticks [6, 16, 18, 28], and spiders [17]. The truncated stop codons are completed by polyadenylation of the mature transcript [18]. The length of the protein-coding genes ranged from 162 bp (*atp8*) to 1659 bp (*nad5*), and their AT/GC contents range from 69.7%/30.3% (*cox1*) to 84.6%/15.4% (*atp8*). Nine of the protein-coding genes (*nad2*, *cox1*, *cox2*, *atp8*, *atp6*, *cox3*, *nad3*, *nad6* and *cytb*) were encoded on the positive strand, while the remaining four (*nad1*, *nad4*, *nad4L* and *nad5*) were encoded on the negative strand.

### Ribosomal RNA genes

Both *ssu rRNA* and *lsu rRNA* genes were found with a length of 700 bp and 1166 bp, respectively similar to other ticks [6, 11, 16, 18, 28]. Their AT/GC compositions were 78.7%/21.3% and 81.6%/18.4% for *ssu rRNA* and *lsu rRNA*, respectively. They were both located on the negative strand between the *tRNA-L2* gene and the CR1 with the *tRNA-V* gene intercalated in between, this organisation follows the other Metastriates [18] (Fig. 1).

### Transfer RNA genes

**Figure 2:**
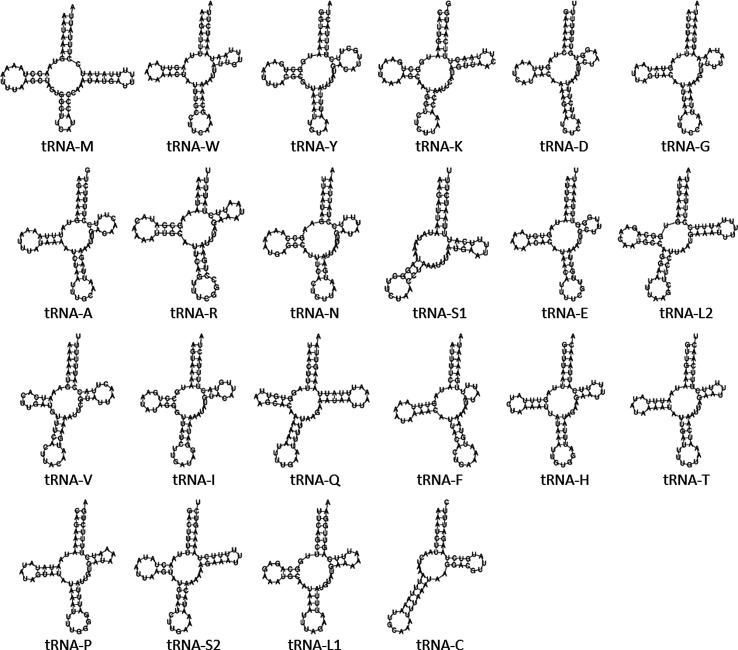
Predicted secondary structure of the mitochondrial *tRNA* genes of *Dermacentor* (*Indocentor*) *auratus* obtained using the Vienna RNA Websuite [10].

Twenty two *tRNA* genes were identified including 2 *tRNA-L* and 2 *tRNA-S* (Figs. 1, 2). Their size ranged from 56 bp (*tRNA-S1*) to 68 bp (*tRNA-M*) (Table 1). The majority of the *tRNA* genes (*tRNA-M*, *tRNA-W*, *tRNA-K*, *tRNA-D*, *tRNA-G*, *tRNA-A*, *tRNA-R*, *tRNA-N*, *tRNA-S1*, *tRNA-E*, *tRNA-I*, *tRNA-T*, *tRNA-S2* and *tRNA-C*) were encoded on the positive strand, while the remaining eight were encoded on the negative strand (Fig. 1). The AT/GC content of the tRNAs ranged from 70.1%/29.9% (*tRNA-K*) to 90.2%/9.8% (*tRNA-G*). Prediction of the tRNA secondary structure showed that 20 of the tRNAs have the standard cloverleaf structure, while tRNA-S1 (anticodon TCT) and tRNA-C were missing the D-arm (Fig. 2). Mitochondrial tRNA-S secondary structure lacking the D-arms is a common feature in most animal species, including ticks [28]. Mitochondrial tRNA-C secondary structures are variable among tick species with some missing D-arm and/or T-arm and some having standard cloverleaf structure; *D. auratus* was only lacking the D-arm, a feature previously observed in other tick species [27, 28] (Fig. 2). The sizes, coding strand and arrangement of the *tRNA* genes were similar to what is observed in the mitogenome of other *Dermacentor* species [11] and other ticks [6, 16, 18, 28].

### Non-coding sequences

Duplicate CRs were found; they contained the regulatory elements of mitochondrion transcription and replication. CR1 and CR2 spanned 311 bp and 304 bp in length, respectively and shared a common identical core sequence of 249 bp (position 9080-8832 for CR1 and 14692-14444 for CR2). Their AT/GC content was 65.9%/34.1% and 66.8%/33.2%, respectively on their full length and 64.3%/35.7% for the core sequence. CR1 was located between *ssu rRNA* and *tRNA-I* and CR2 between *tRNA-L1* and *tRNA-C*, both on the negative strand. These locations have been observed in many other Metastriates [6,16,18,28]. Three non-coding motifs called “tick-boxes” were identified, a number identical to what was reported in Metastriates [18]. They are short post-transcriptional regulatory elements of 18 bp [18]. Tick-Boxes 1 and 2 shared an identical sequence, while Tick-Box 3 showed only 1 nucleotide difference. Tick-Box 1 was located on the negative strand at the 3′ end of the *nad1* gene, a position widely overlooked in many published tick mitochondrial genomes where the termination of the *nad1* gene is extended to a full TAA stop codon and abuts or overlaps with the *tRNA-E* gene incorporating Tick-Box 1, as pointed out by [18]. Tick-Box 2 was located between the *lsu RNA* and the *tRNA-L2* genes on the negative strand, while Tick-Box 3 was located between *tRNA-Q* and *tRNA-F* on the positive strand, following the general organisation of the other Metastriates [18].

### Phylogenetic analysis

**Figure 3:**
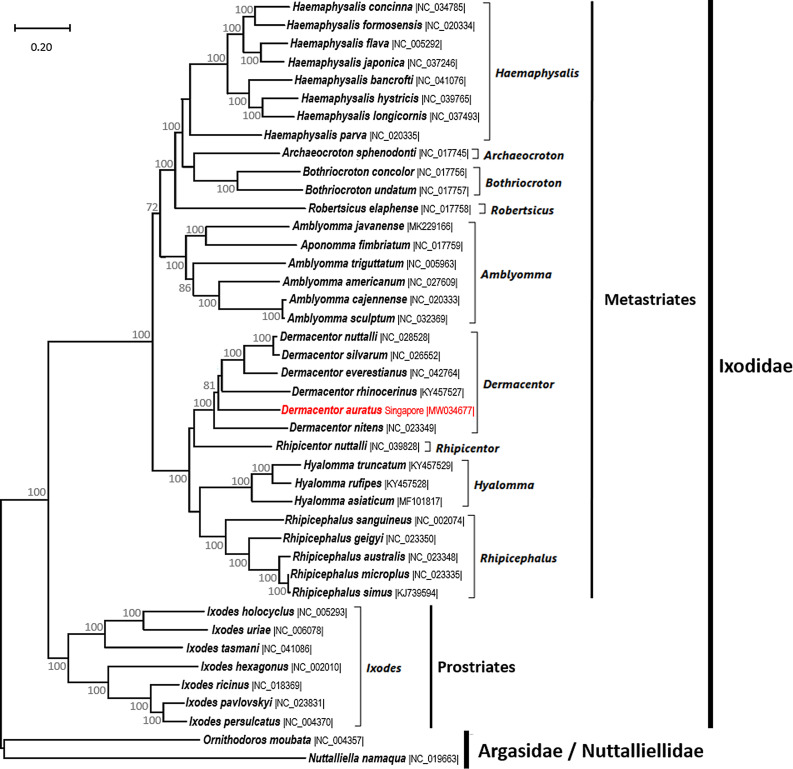
Maximum likelihood phylogeny inferred on the mitochondrial genomes of *Dermacentor* (*Indocentor*) *auratus* obtained in this study (MW034677) (highlighted) and 41 other tick mitochondrial genomes retrieved from GenBank. Accession numbers of the sequences are provided between vertical bars. The mitogenomes of the Argasidae and Nuttalliellidae were used as outgroups to root the tree. The ML analysis used the GTR + Γ + I model of evolution [26]. Reliability of tree topology, branch support and nodal robustness were assessed by non-parametric bootstrap using 1000 replicates (only >70% shown). Analysis performed with MEGA X [12].

The ML analysis was performed on the mitochondrial genomes of *D. auratus* obtained in this study (MW034677) and 39 other Ixodidae, as well as 1 Argasidae and 1 Nuttalliellidae used as outgroups to root the tree (Fig. 3). The phylogenetic tree of the Ixodidae was strongly supported with the major genera clearly separated (bootstrap values 100%) (Fig. 3) and was monophyletic, following the recent establishment of the genera *Robersticus* and *Archaeocroton* to accommodate *Amblyomma elaphense* and *Amblyomma sphenodonti* [2], which previously had controversial phylogenetic positions (Fig. 3) [2, 6, 16, 28]. Expectedly, *D. auratus* is clustered among the other *Dermacentor* species (Fig. 3).

### *Dermacentor* subgenera

Up to seven subgenera were recognised within the genus *Dermacentor*: *Dermacentor*, *Indocentor*, *Asiacentor*, *Americentor*, *Serdjukovia*, *Kohlsiella* and *Olenvenia*, with the validity of the last three remaining controversial [29]. The type species of the subgenus *Indocentor* is *D. auratus.* The mitochondrial genome of *D. auratus* obtained here constitutes a useful reference for future studies on *Dermacentor* subgeneric classification. This will become particularly true when more sequences from different species become available, and reaching this point could be accelerated with the use of the simple protocol adopted here.

## Conclusion

This study reports the complete mitochondrial genome of *D.* (*Indocentor*) *auratus* obtained using a simple method of amplification and sequencing based on two long-range PCRs and next generation sequencing. The mitogenome of *D. auratus* is circular and has the typical Metastriates tick mitochondrial genome organisation. All the genes were annotated and the secondary structure of the tRNAs was determined. Phylogenetic analyses indicated that *D. auratus* clustered with other *Dermacentor* species. The availability of the complete mitochondrial genome sequence of *D. auratus* will provide a useful reference to help in the rapid and accurate identification of this important vector of TBDs, as well as for further studies on the phylogeny and evolution history of hard ticks.
